# Persistently higher serum sCD40L levels are associated with outcome in septic patients

**DOI:** 10.1186/s12871-021-01241-9

**Published:** 2021-01-22

**Authors:** Yingjian Liang, Chengrui Zhu, Yini Sun, Zhiliang Li, Liang Wang, Yina Liu, Xin Li, Xiaochun Ma

**Affiliations:** Department of Critical Care Medicine, The First Hospital of China Medical University, North Nanjing Street 155, Shenyang, 110001 Liaoning Province China

**Keywords:** Soluble CD40 ligand, Sepsis, Mortality

## Abstract

**Background:**

Soluble CD40 ligand (sCD40L) exhibits proinflammatory and procoagulant effects. Recent data indicated that sCD40L plays a significant role in septic patients. The aim of the present study was to determine sCD40L changes in surgical patients without sepsis (SWS) and surgical sepsis patients (SS) during the first 3 days after intensive care unit (ICU) admission and to observe the association between sCD40L and mortality.

**Methods:**

Time changes in sCD40L levels were assessed for 3 days after ICU admission in 49 patients with SS and compared with those in 19 SWS patients. Serum sCD40L concentration was detected by ELISA. Survival at 28 days served as the endpoint.

**Results:**

SS had significantly higher sCD40L levels than SWS and control patients. We observed an association between sCD40L levels ≥1028.75 pg/mL at day 2 and 28-day mortality (odds ratio = 7.888; 95% confidence interval = 1.758 to 35.395; *P* = 0.007). We could not discover any significant differences in sex, presence of septic shock, site of infection, length of stay in the ICU, PaO_2_/FiO_2_ ratio, incidence of AKI, ARDS, or type of surgery between nonsurvivors and survivors.

**Conclusions:**

Septic patients show persistently higher circulating sCD40L levels in the first 3 days after ICU admission, and serum sCD40L levels are associated with the mortality of patients with sepsis. Thus, serum sCD40L may be used as a reliable biomarker and therapeutic target in sepsis.

**Supplementary Information:**

The online version contains supplementary material available at 10.1186/s12871-021-01241-9.

## Background

Sepsis is the overwhelming inflammatory host response to the infectious agent causing the overexpression of inflammatory mediators [1]. Moreover, sepsis is also generally associated with coagulation abnormalities and presents as thromboembolic disease or clinically less apparent microvascular fibrin deposition [2]. In sepsis, inflammation and coagulation are cross-linked, and inflammation causes coagulation activation. In contrast, coagulation also affects the activation of inflammation, which promotes disease progression and leads to organ dysfunction [3].

CD40 ligand (CD40L) and the soluble form of CD40L (sCD40L) are members of the tumor necrosis factor (TNF) family and are expressed in a variety of cell types, including B cells, epithelial cells, fibroblasts, endothelial cells (ECs), and platelets. CD40L and sCD40L exhibit proinflammatory and procoagulant effects [4, 5]. Previous studies have found that serum CD40L levels are elevated in patients with sepsis and are associated with mortality in these patients [6–9].

In surgical patients, endothelial cell damage, inflammation, and coagulation changes occurred after surgery. Therefore, the purpose of this study was to determine sCD40L changes in surgical patients without sepsis (SWS) and in surgical sepsis patients (SS) during the first 3 days after ICU admission and to observe the relationship between sCD40L and mortality.

## Methods

### Design and subjects

This was a prospective, observational study. Sixty-eight patients who underwent abdominal surgery were enrolled in the Intensive Care Unit of the First Affiliated Hospital, China Medical University from October 1, 2013 to February 28, 2014. These patients included 49 SS and 19 SWS. Six healthy controls were selected. The Ethical Committee of the First Affiliated Hospital of China Medical University approved this study. Because all the patients in our study were admitted to ICU with endotracheal intubation after general anesthesia, these patients were unconscious and unable to sign informed consent due to the influence of sedatives and analgesics. Thus, informed consent forms were signed by the patients’ family members (No. 2015–113-2).

Forty-nine SS patients had abdominal infections or pulmonary infections in addition to other sites of infection. Sepsis and septic shock were diagnosed according to the Surviving Sepsis Campaign guidelines committee 2012 [10]. The exclusion criteria were age < 18 years, pregnancy, lactation, human immunodeficiency virus (HIV) positive, hematological tumor, immunosuppressed, or undergoing steroid or radiation therapy.

### Variables recorded

In all the patients, age, sex, Acute Physiology and Chronic Health Evaluation II (APACHE II) score, Sepsis-related Organ Failure Assessment (SOFA) score, sCD40L, International Society on Thrombosis and Haemostasis (ISTH) score, Japanese Association for Acute Medicine (JAAM) score, platelets, prothrombin time international normalized ratio (PT-INR), activated partial thromboplastin time (aPTT), fibrinogen, D-dimer, fibrinogen degradation product (FDP), leukocytes, lactate, site of infection, length of stay in the ICU, number of affected organs, acute kidney injury (AKI), acute respiratory distress syndrome (ARDS), pressure of arterial oxygen/fraction inspired oxygen (PaO_2_/FiO_2_) ratio, sepsis-induced liver injury, invading organisms, and culprit organ with primary sepsis were recorded. Survival at 28 days was used as the study endpoint.

### Blood samples

For patients, blood samples were drawn on days 1, 2 and 3 after ICU admission. For healthy controls, fasting blood samples were obtained at 8:00 am. Serum blood samples were centrifuged at 1500 g for 15 min. The temperature was maintained at 4 °C in all steps after blood collection. Aliquots were stored at − 80 °C for further analysis. Samples were thawed only once. Blood samples from each patient with SS were cultured and analyzed to identify the pathogenic bacteria.

### Soluble CD40L determination

Serum sCD40L concentration was detected by enzyme-linked immunosorbent assays (ELISA) from Life Science, Inc. (China). Each sample was measured in duplicate. The detection limit was 6.1 pg/mL. The intratest variability among the duplicates of all samples was less than 10%.

### Statistical analysis

Continuous variables were analyzed as medians and interquartiles (IQ) 25 and 75% (Q25-Q75), and categorical variables were analyzed as frequencies and percentages. Significant differences in continuous variables between groups were analyzed using the Mann-Whitney U test, and categorical variables were calculated using the chi-square test and Fisher’s exact test, as appropriate. Receiver operating characteristic (ROC) curves were used to analyze serum sCD40L levels at days 1, 2 and 3 and establish cut off values. Survival analysis was performed with Kaplan-Meier method curves. Multiple logistic regression analysis was applied to predict 28-day mortality. To determine the association between sCD40L serum levels and other continuous variables at days 1, 2 and 3, Spearman’s rank correlation coefficient was used. All *P*-values < 0.05 were considered statistically significant. SPSS 20.0 (SPSS Inc., Chicago, IL, USA) was used for all statistical analyses.

## Results

### Patient characteristics

Baseline characteristics of 49 SS, 19 SWS and 6 age- and sex-matched healthy controls are shown in Table 1. The levels of sCD40L in healthy controls were 469.50 (406.83–547.36) pg/mL, and the sCD40L levels in SWS and SS were significantly increased. The difference between the two groups was statistically significant (Table 1, *P* = 0.000 vs healthy controls). Compared with SWS, SS also exhibited increased APACHE-II (*P* = 0.001), SOFA (*P* < 0.001), ISTH (*P* < 0.001), and JAAM (*P* < 0.001) scores; AKI (*P* = 0.000), ARDS (*P* = 0.000), PT-INR (*P* = 0.001), fibrinogen (*P* = 0.022), FDP (*P* < 0.001), D-dimer (*P* < 0.001), and lactate (*P* = 0.006) levels; 28-day mortality (*P* = 0.001), longer length of stay in the ICU, and lower PaO_2_/FiO_2_ ratio (Table 1). However, type of surgery did not differ between the SS and SWS groups.

**Table 1:** Baseline demographic and clinical characteristics in the studied population group

	Healty control *n* = 6	SS *n* = 19	SWS *n* = 49	*P*
Sex (male/female)	3/3	12/7	34/15	0.586
Age (years)-median (p25-p75)	53.00 (42.25–62.00)	76.00 (71.00–81.00)	61.00 (45.00–73.00)	0.005
sCD40L(pg/ml)-median (p25-p75)	469.50 (406.83–547.36)	831.36 (526.58–981.72)	1022.68 (554.85–2215.13)	0.000
APACHE II score-median (p25-p75)		9.00 (7.00–11.00)	14.00 (11.00–16.00)	0.001
SOFA score-median (p25-p75)		1.00 (0.00–2.00)	8.00 (6.00–10.00)	0.000
ISTH score-median (p25-p75)		0.00 (0.00–2.00)	3.00 (2.50–4.50)	0.000
JAAM score-median (p25-p75)		1.00 (0.00–2.00)	4.00 (3.00–5.00)	0.000
PT-INR-median (p25-p75)		1.16 (1.12–1.34)	1.41 (1.21–1.66)	0.001
aPTT (seconds)-median (p25-p75)		41.10 (36.10–81.40)	49.40 (41.55–60.85)	0.080
Fibrinogen (g/L)-median (p25-p75)		3.10 (1.96–3.60)	3.94 (2.50–5.88)	0.022
FDP (ug/dl)-median (p25-p75)		5.46 (3.60–13.42)	21.81 (12.24–48.66)	0.000
D-Dimer (ug/ml)-median (p25-p75)		1.71 (0.80–3.58)	4.81 (3.24–10.11)	0.000
Platelet-median*10^3^/mm^3^ (p25-p75)		157.00 (136.00–191.00)	136.00 (77.00–214.50)	0.305
Leukocytes-median*10^3^/mm^3^ (p25-p75)		7.87 (5.28–11.90)	10.62 (7.50–15.44)	0.030
Lactate (mmol/L)-median (p25-p75)		1.50 (0.90–1.80)	2.00 (1.30–3.90)	0.006
28 days mortality (%)		0/19 (0)	20/49 (40.82)	0.000
Length of stay in the ICU (days)-median (p25-p75)		3.74 (3–5)	21.57 (13–28)	0.000
AKI-n (%)		0/19 (0)	16/49 (32.65)	0.000
Sepsis-induced liver injury-n (%)		0/19 (0)	3/49 (6.12)	0.277
ARDS-n (%)		0/19 (0)	21/49 (42.86)	0.000
PaO_2_/FiO_2_ ratio-median(p25-p75)		318.9 (300–330)	234.1 (170–300)	0.000
Subtotal gastrectomy-n(%)		3/19 (15.79)	10/49 (20.41)	0.549
Enteroanastomosis-n(%)		8/19 (42.11)	19/49 (38.77)	0.805
Cholecystectomy-n(%)		3/19 (15.79)	8/49 (16.33)	0.958
Choledocholithotomy-n(%)		5/19 (26.31)	12/49 (24.49)	0.878

*SWS* Surgical patients without sepsis, *SS* Surgical sepsis patients, *p 25–75* Percentile 25th–75th, *APACHE II* Acute Physiology and Chronic Health Evaluation II, *SOFA* Sepsis-related Organ Failure Assessment, *ISTH* International Society on Thrombosis and Haemostasis, *JAAM* Japanese Association for Acute Medicine, *PT-INR* Prothrombin time international normalized ratio, *aPTT* Activated partial thromboplastin time, *FDP* Fibrinogen degradation product, *AKI* Acute kidney injury, *ARDS* Acute respiratory distress syndrome, *PaO*_*2*_*/FiO*_*2*_ Pressure of arterial oxygen/fraction inspired oxygen

A total of 27 strains were isolated from the blood samples of 20 patients in the SS group, including seven strains with methicillin-resistant coagulase-negative *Staphylococcus*; five with *Acinetobacter baumannii*; five with non-*Candida albicans*; three with *Enterococcus faecium*, two each with methicillin-resistant *Staphylococcus aureus*, *Pseudomonas aeruginosa*, and *Klebsiella pneumoniae*; and one with *Escherichia coli*. More than two strains were isolated from the blood samples of 8 patients.

### Demographic and clinical characteristics of nonsurvivors and survivors in surgical sepsis patients

In total, 20 SS patients died, and 29 patients survived. The demographic and clinical features of these patients are shown in Table 2 and Supplemental Table 1. Nonsurviving patients had higher serum sCD40L (*P* = 0.009) and aPTT than surviving patients at day 2 (*P* = 0.020) and higher lactate at day 3 (*P* = 0.001). In addition, older age (*P* = 0.023) was observed in the group of nonsurviving patients compared with the group of surviving SS patients. No significant differences in sex, presence of septic shock, site of infection, length of stay in the ICU, PaO_2_/FiO_2_ ratio, incidence of AKI, ARDS, or type of surgery were noted between nonsurvivors and survivors. In addition, on days 1, 2, and 3 after ICU admission, no statistically significant differences in APACHE II score, SOFA score, ISTH score, JAAM score, PT-INR, fibrinogen, FDP, D-dimer, platelet and leukocytes were noted between the nonsurviving and surviving groups of SS.

**Table 2:** Biochemical characteristics of survivor and nonsurvivor surgical sepsis patients on day 2 of ICU admission

	Survivor (*n* = 29)	Non-survivor (*n* = 20)	*P*
Gender (male/female)	22/7	12/8	0.236
Age (years)-median(p25-p75)	56.00 (31.50–64.00)	71.00 (52.25–77.25)	0.023
Septic shock− n (%)	17 (58.6)	10 (50.0)	0.551
Site of infection			0.508
Respiratory − n (%)	5 (17.2)	5 (25.0)	
Abdominal − n (%)	24 (82.8)	15 (75.0)	
APACHE II score-median(p25-p75)	12.00 (8.00–16.50)	14.00 (11.25–17.00)	0.156
SOFA score-median(p25-p75)	6.00 (5.00–10.00)	8.50 (6.00–10.00)	0.216
ISTH score-median(p25-p75)	4.00 (2.00–5.00)	3.00 (3.00–4.75)	0.901
JAAM score-median(p25-p75)	4.00 (2.50–5.00)	4.00 (2.50–4.75)	0.835
PT-INR-median(p25-p75)	1.36 (1.25–1.72)	138.5 (128.25–170.75)	0.760
aPTT (seconds)-median(p25-p75)	51.00 (43.60–66.95)	68.65 (53.20–113.95)	0.020
Fibrinogen(g/L)-median(p25-p75)	3.74 (2.55–6.70)	2.83 (1.70–4.78)	0.106
FDP (ug/dl)-median(p25-p75)	16.96 (10.43–33.01)	20.40 (7.37–32.33)	0.823
D-Dimer (ug/ml)-median(p25-p75)	3.96 (2.95–7.61)	3.86 (2.19–7.86)	0.502
Platelet- median*10^3^/mm^3^ (p25-p75)	109.00 (47.00–191.00)	124.50 (65.25–174.25)	0.903
Leukocytes-median*10^3^/mm^3^(p25-p75)	13.71 (6.83–17.93)	13.91 (9.06–19.22)	0.376
Lactate (mmol/L)-median (p25-p75)	2.10 (1.30–3.15)	2.75 (1.65–5.60)	0.203
sCD40L(pg/ml)-median (p25-p75)	747.21 (422.46–981.47)	1214.72 (696.28–2089.57)	0.009
Length of stay in the ICU (days)-median (p25-p75)	17.62 (5–20)	12.55 (7–19)	0.341
AKI –n (%)	10/29 (34.48)	6/20 (30.00)	0.749
ARDS-n (%)	12/29 (41.38)	9/20 (45.00)	0.806
PaO_2_/FiO_2_ ratio-median(p25-p75)	232.9 (170–300)	236.0 (170–310)	0.900
Subtotal gastrectomy-n(%)	5/29 (17.24)	5/20 (25.00)	0.518
Enteroanastomosis-n(%)	11/29 (37.93)	8/20 (40.00)	0.889
Cholecystectomy-n(%)	5/29 (17.24)	3/20 (15.00)	0.839
Choledocholithotomy-n(%)	8/29 (27.59)	4/20 (20.00)	0.554

*p25–75* Percentile 25th–75th, *APACHE II* Acute Physiology and Chronic Health Evaluation II, *SOFA* Sepsis-related Organ Failure Assessment, *ISTH* Thrombosis and Haemostasis, *JAAM* Japanese Association for Acute Medicine, *PT-INR* Prothrombin time international normalized ratio, *aPTT* Activated partial thromboplastin time, *FDP* Fibrinogen degradation product, *AKI* Acute kidney injury, *ARDS* Acute respiratory distress syndrome, *PaO*_*2*_*/FiO*_*2*_ Pressure of arterial oxygen/fraction inspired oxygen

### Predictive factors for 28-day mortality

**Fig. 1:**
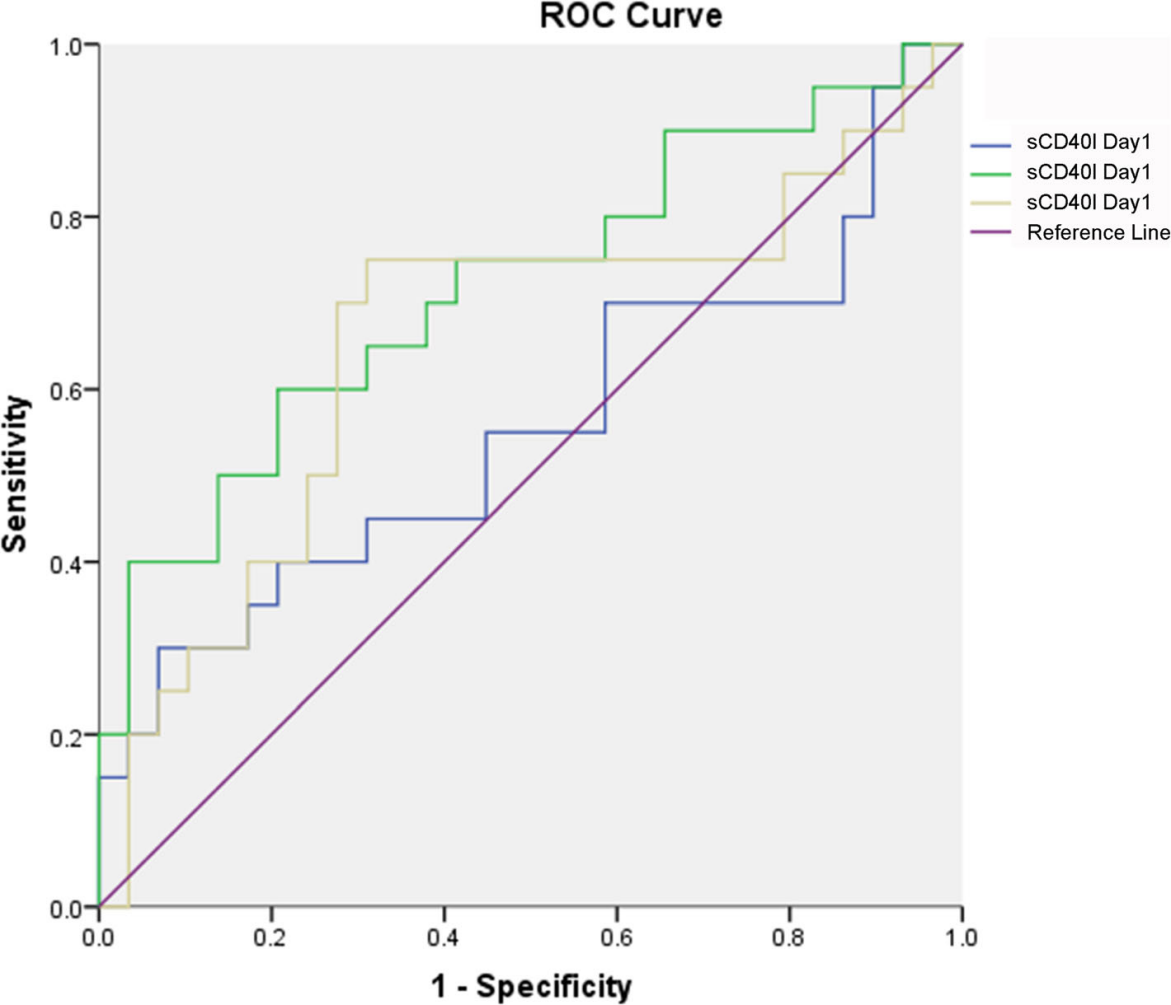
Receiver operation characteristic analysis using sCD40L levels ≥1028.75 pg/mL at day 2 as a 28-day mortality predictor

**Fig. 2:**
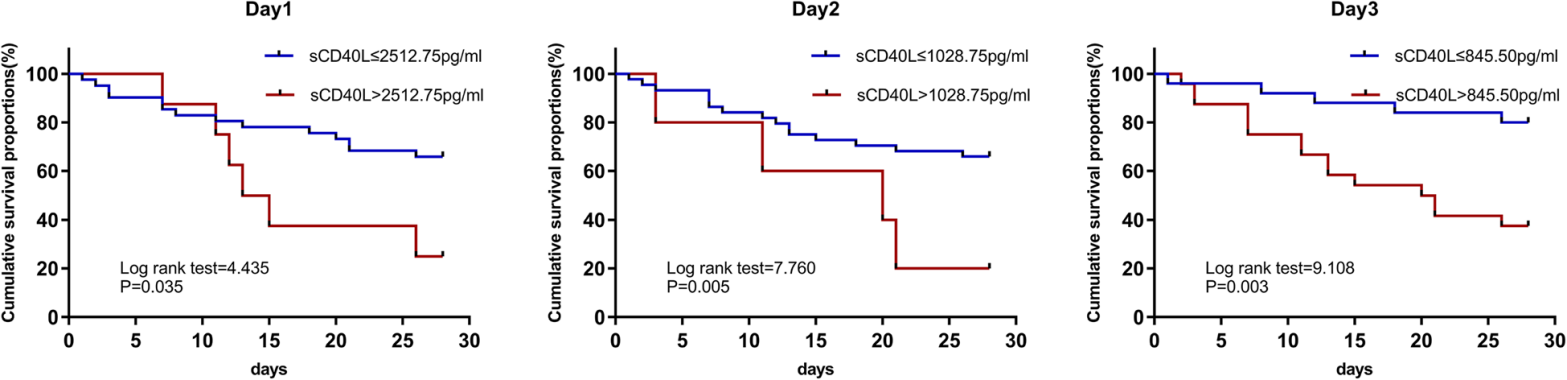
Survival curves at 28 days according to serum sCD40L levels at days 1, 2 and 3 in surgical sepsis patients admitted to the ICU

The area under the curve (AUC) for serum sCD40L levels at day 2 (95% confidence interval (CI) = 0.570–0.871, *P* = 0.009) could predict mortality at 28 days, and the sensitivity and specificity approached 60.0 and 79.3%, respectively (Fig. 1). Kaplan-Meier survival analysis showed that patients with higher serum sCD40L levels at day 1 (*P* = 0.035), day 2 (*P* = 0.005), and day 3 (*P* = 0.003) had a higher risk of death at 28 days than patients with lower levels (Fig. 2). In addition, patient age ≥ 65 years (odds ratio = 7.929; 95% CI = 1.809 to 34.750; *P* = 0.006) and serum sCD40L levels at day 2 ≥ 1028.75 pg/mL (odds ratio = 7.888; 95% CI = 1.758 to 35.395; *P* = 0.007) were significant predictive factors for 28-day mortality in multiple logistic regression analysis.

### Association between sCD40L levels and other clinical parameters in patients with surgical sepsis

In addition, no relationship was observed between serum sCD40L levels and APACHE II, SOFA, ISTH, and JAAM score as well as PT-INR, fibrinogen, FDP, D-dimer, leukocyte, platelets and lactate levels in the SS group on days 1, 2, and 3 after ICU admission (Table 3).

**Table 3:** Association between sCD40L levels and other clinical parameters in patients with surgical sepsis

	sCD40L day1		sCD40L day2		sCD40L day3	
	Rho	P	Rho	P	Rho	P
APACHE II score	−0.215	0.138	−0.045	0.761	−0.208	0.151
SOFA score	−0.158	0.279	−0.073	0.617	0.069	0.639
ISTH score	0.016	0.914	−0.051	0.726	0.098	0.501
JAAM score	−0.016	0.916	0.016	0.912	0.216	0.136
PT-INR	0.146	0.318	0.166	0.255	0.055	0.708
aPTT	0.132	0.367	−0.027	0.851	−0.043	0.769
Fibrinogen	−0.092	0.529	−0.065	0.655	−0.202	0.163
FDP	0.131	0.370	0.137	0.347	0.085	0.562
D-Dimer	0.171	0.240	0.077	0.600	0.069	0.636
Platelet	0.046	0.752	0.122	0.405	−0.003	0.983
Leukocytes	0.223	0.124	0.158	0.277	0.061	0.679
Lactate	−0.050	0.733	0.194	0.181	0.111	0.447

*APACHE II* Acute Physiology and Chronic Health Evaluation II, *SOFA* Sepsis-related Organ Failure Assessment, *ISTH* Thrombosis and Haemostasis, *JAAM* Japanese Association for Acute Medicine, *PT-INR* Prothrombin time international normalized ratio, *aPTT* Activated partial thromboplastin time, *FDP* Fibrinogen degradation product

## Discussion

It can be concluded from this study that serum sCD40L levels in SS patients persistently increased significantly in the first 3 days after admission to the intensive care unit, and circulating sCD40L levels were increased on the second day of admission in the nonsurviving group compared with the surviving group. A novel finding of this study was that SWS patients also exhibited a slight increase in sCD40L, but the level was not as great as that noted in SS patients.

Several studies have reported that sCD40L levels can predict the prognosis of patients with sepsis [8, 11]. In our study, we found that serum sCD40L levels ≥1028.75 pg/mL at day 2 were associated with a higher death risk during the 28-day period in the multiple logistic regression analysis. Serum sCD40L levels could be used as a 28-day mortality biomarker. However, we did not find a relationship between sCD40L levels and sepsis severity criteria, such as APACHE II score and SOFA score. We only observed higher lactatemia in nonsurviving compared with surviving SS patients at day 3 after admission. Serum blood samples were obtained at ICU admission, but APACHE II or SOFA scores were calculated 24 h after admission to the ICU. We were not sure if this time gap affects the association between the two variables.

sCD40L has a dual prothrombotic and proinflammatory role. sCD40L connects to circulating monocytes through its receptor CD40, promoting their adhesion to the vascular endothelium. sCD40L also binds to CD40 on endothelial cell surfaces. Studies have shown that sCD40L stimulates its own expression by interacting with CD40 on the surface of these cells [12]. In sepsis, EC activation induced adhesion receptors and released inflammatory mediators, such as interleukin (IL)-1, IL-6 and tumor necrosis factor [13, 14]. sCD40L also affects the neutrophil oxidative burst and neutrophil extracellular trap [5, 15]. Previous studies and our studies suggested that sCD40L exhibits no correlation with other coagulation factors except tissue factor (TF) [8]. The main reason was that activated ECs initiate the exogenous coagulation pathway by upregulating TF and downregulating the expression of thrombomodulin [16], favoring a local procoagulant status. In experimental models, sCD40L enhanced platelet activation and aggregation and induced thrombus formation [17]. All these effects contribute to the development of organ dysfunction and death [18]. After intraoperative operation, ECs were damaged, and the body produced a stress response and inflammatory factors [19]. Therefore, the sCD40L level was also increased after surgery in non-sepsis patients. In sepsis patients, EC damage, intravascular microthrombus formation and production of inflammatory factors are more obvious, and sCD40L levels are much higher than those in patients who undergo simple surgery.

We did not find an association between serum sCD40L levels and platelet counts, although 95% sCD40L was derived from platelets [20]. CD40L is stored in α-granules in unstimulated platelets, undergoes conformational changes during platelet activation, migrates to the surface of platelets and is released into the blood [21]. Soluble CD40L enhances platelet activation, aggregation, and platelet-leukocyte conjugation. Therefore, sCD40L was implicated in platelet activation [12]. By interacting with ECs, activated platelets play a key role in inflammatory and procoagulant responses to a pathogen [22].

There were some limitations in our study. First, the sample size was relatively small. Second, the subjects were obtained from a single center. Third, we only determined sCD40L levels for 3 days after ICU admission but did not observe it for a week or longer; thus, we were unable to better conclude the time course of serum sCD40L levels in sepsis patients. Finally, an association between sCD40L levels and the activation function of platelets has been reported; we did not examine markers of platelet activation to analyze the relationship with sCD40L levels.

## Conclusions

Septic patients show persistently higher circulating sCD40L levels in the first 3 days after ICU admission, and serum sCD40L levels are associated with the mortality of patients with sepsis. Thus, serum sCD40L levels may be used as a reliable biomarker and therapeutic target in sepsis.

## Supplementary Information

**Additional file 1: Supplemental Table 1.** Biochemical characteristics of survivor and nonsurvivor surgical sepsis patients on days 1 and 3 after ICU admission.

## Data Availability

The datasets used and/or analysed during the current study are available from the corresponding author on reasonable request.

## Abbreviations

CD40L: CD40 ligand
sCD40L: Soluble CD40 ligand
TNF: Tumor necrosis factor
ECs: Endothelial cells
SWS: Surgical patients without sepsis
SS: Surgical sepsis patients
APACHE II: Acute physiology and chronic health evaluation II
SOFA: Sepsis-related organ failure assessment
ISTH: International society on thrombosis and haemostasis
JAAM: Japanese association for acute medicine
PT-INR: Prothrombin time international normalized ratio
aPTT: Activated partial thromboplastin time
FDP: Fibrinogen degradation product
AKI: Acute kidney injury
ARDS: Acute respiratory distress syndrome
PaO_2_/FiO_2_: Pressure of arterial oxygen/fraction inspired oxygen
