# Supplementary material for: Persistently higher serum sCD40L levels are associated with outcome in septic patients
Source: BMC Anesthesiol. 2021 Jan 22;21:26. doi: 10.1186/s12871-021-01241-9 (PMC7820820; doi:10.1186/s12871-021-01241-9)
Supplement: Supplementary file 1 — Additional file 1: Supplemental Table 1. Biochemical characteristics of survivor and nonsurvivor surgical sepsis patients on days 1 and 3 after ICU admission. [file 12871_2021_1241_MOESM1_ESM.docx]

**Supplementary Table 1**, Biochemical characteristics of survivor and nonsurvivor surgical sepsis patients on day 1 and day 3 at ICU admission

| Variables | Day 1 |  |  |  | Day 3 | |  | |  | |
| --- | --- | --- | --- | --- | --- | --- | --- | --- | --- | --- |
|  | Survivor(n=29) | Non-survivor(n=20) | *P* |  | Survivor(n=29) | | Non-survivor(n=20) | | *P* | |
| APACHE Ⅱ score | 13.00(11.00-16.00) | 14.50(11.00-16.50) | 0.653 | 11.00(8.00-14.00) | | 12.00(11.00-17.00) | | 0.126 | |  |
| SOFA score | 7.00(6.00-10.50) | 8.00(6.00-9.00) | 0.821 | 6.00(5.00-9.00) | | 7.50(6.25-10.75) | | 0.051 | |  |
| ISTH score | 4.00(3.00-4.50) | 3.00(2.00-4.75) | 0.228 | 3.00(2.00-4.00) | | 3.00(2.00-4.75) | | 0.788 | |  |
| JAAM score | 5.00(3.00-5.00) | 4.00(2.25-4.75) | 0.135 | 4.00(3.00-5.00) | | 4.00(3.00-5.00) | | 0.942 | |  |
| PT-INR | 1.41(1.24-1.63) | 1.42(1.19-1.66) | 0.935 | 1.33(1.16-1.51) | | 1.36(1.21-1.56) | | 0.555 | |  |
| aPTT | 45.50(39.75-60.50) | 55.85(45.68-64.33) | 0.075 | 53.40(45.10-73.15) | | 63.05(51.58-141.08) | | 0.065 | |  |
| fibrinogen | 4.69(2.83-6.20) | 3.02(2.32-4.80) | 0.125 | 4.23(3.54-6.69) | | 3.13(2.38-5.50) | | 0.066 | |  |
| FDP | 24.54(12.89-46.59) | 19.63(9.41-50.55) | 0.647 | 24.45(12.90-39.57) | | 17.47(6.71-30.15) | | 0.143 | |  |
| D-Dimer | 4.92(3.58-9.69) | 4.33(2.41-11.22) | 0.464 | 5.22(3.55-9.22) | | 3.70(2.37-7.50) | | 0.163 | |  |
| platelet | 135.00(59.50-234.00) | 142.50(98.50-191.25) | 0.831 | 103.00(63.50-208.00) | | 86.50(35.25-150.25) | | 0.189 | |  |
| leukocytes | 12.80(7.59-16.87) | 10.42(7.03-13.19) | 0.382 | 12.30(8.36-19.95) | | 14.51(11.28-21.33) | | 0.502 | |  |
| lactate | 1.70(1.20-3.60) | 2.40(1.60-4.25) | 0.151 | 1.20(1.00-2.05) | | 3.25(1.63-5.43) | | 0.001 | |  |
| sCD40L | 1007.78  (556.15-1896.91) | 1259.84  (461.60-2721.72) | 0.502 | 558.21(398.20-1188.67) | | 1146.48  (502.25-2082.24) | | 0.067 | |  |

APACHE II = Acute Physiology and Chronic Health Evaluation II; SOFA =Sepsis-related Organ Failure Assessment; ISTH =Thrombosis and Haemostasis , JAAM =Japanese Association for Acute Medicine; PT-INR =prothrombin time international normalized ratio; aPTT=activated partial thromboplastin time; FDP=Fibrinogen degradation product
